# Type V Pouch Colon, Prune Belly Syndrome, and Congenital Anterior Urethrocutaneous Fistula

**DOI:** 10.21699/jns.v6i2.481

**Published:** 2017-04-15

**Authors:** Prince Raj, Hirendra Birua

**Affiliations:** Department of Pediatric Surgery, Rajendra Institute of Medical Sciences, Ranchi

**Keywords:** Anorectal malformations, Congenital pouch colon, Congenital urethrocutaneous fistula, Segmental dilatation of colon.

## Abstract

Congenital pouch colon (CPC) or short colon syndrome is a rare type of anorectal malformation(ARM). Type V is the rarest form of CPC. We present a 1-day-old male child with type V CPC with prune belly syndrome and congenital anterior urethrocutaneous fistula (CAUF).

## CASE REPORT

**Figure 1:**
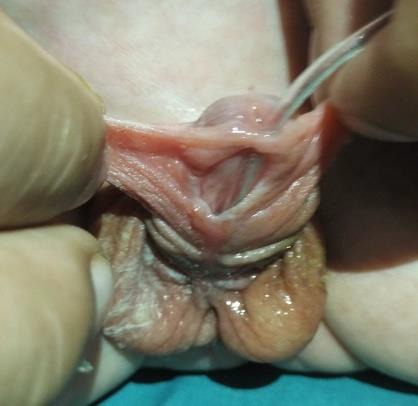
Urethrocutaneous fistula.

A full-term male baby, third in birth order, born by vaginal delivery to a 24 year mother, was brought to our hospital with an absent anal opening and hugely distended abdomen on day 1 of life. Antenatal period was unsupervised. On examination, the abdomen was distended and flabby, with visible loops of intestine and peristaltic movement. The scrotum was empty, and testes were impalpable on both sides. The perineal examination revealed absent anal opening and well formed median raphe with a flat perineum. Phallus was flat with a large urethrocutaneous fistula on ventral side (Fig.1). There was no chordee and dorsal hooded prepuce. Penoscrotal transposition was present. A plain abdominal and a cross table prone lateral radiograph showed a large air fluid level covering more than half of the width of the abdomen and crossing the midline, suggestive of a high anorectal anomaly with pouch colon. An abdominal ultrasonogram showed a distended urinary bladder and bilateral grossly hydronephrotic kidneys, with bilateral dilated and tortuous ureters and distended bowel loops. Initial hematological investigation showed normal renal parameters, hemogram and electrolytes. After initial resuscitation, colostomy was planned. Exploration revealed no muscular tissue in the abdominal wall except for the fascial layer. Recto sigmoid was replaced by a pouch like structure with a thick and wide colovesical fistula. There was an abrupt transition from the normal caliber proximal bowel to the distal dilated pouch, it lacked haustrations, appendices epiploicae, and taenia coli. There was also segmental dilatation of colon proximal to the normal intervening colon (Fig.2). The bladder was distended, and both the ureters were dilated and tortuous. The colovesical fistula was disconnected, the pouch colon was excised, and proximal colon was brought out and end colostomy was done. Segmental dilated colon was left as such to reassess in the second stage. Postoperative recovery was uneventful. Histopathological examination of the resected colonic pouch showed normal colonic mucosa and ganglion cells. In view of the normal renal function and spontaneously emptying bladder completely without any obstruction, watchful waiting was planned, and the patient was put on prophylactic antibiotics. Baby is currently on regular follow up with a plan to do abdomino-perineal pull through of colon and repair the CAUF at later date.

## DISCUSSION

Type V is the rarest form of CPC comprising double pouch with short intervening segment of normal colon [3]. But there are other variants of CPC reported in literature in last one decade which does not fit into either of the classification proposed and thus it was suggested that type V CPC should be added to the original Narashima Rao classification which would include all the other variants of CPC, like CPC with segmental dilatation of colon, CPC with PBS, CPC with rectal atresia etc.[1].

CAUF itself is a rare anomaly with less than 50 reported cases so far [2]. Majority of cases are isolated but association of CAUF with other anomalies including ARM is well documented. For cases associated with ARM, it has been postulated that the defect in the urethral plate which is the ventral extension of cloacal plate, may affect the cloacal membrane as well [3]. Though other mechanism may also play a role in view of associated PBS in our patient where rupture of megalourethra may be a possibility.

Management of isolated CAUF is not difficult and has good outcome post surgical repair, but in complex cases where it is a part of ARM and PBS, careful planning is required. Herein, type V CPC takes priority where the distal dilated pouch is excised with closure of recto-vesical with creation of end colostomy. Proximal dilated colon may be left in-situ to be assessed in second stage where it may come to the normal caliber and may be utilized in pull through[4].

## Footnotes

**Source of Support:** None

**Conflict of Interest:** None
